# Native Phytoremediation Potential of *Urtica dioica* for Removal of PCBs and Heavy Metals Can Be Improved by Genetic Manipulations Using Constitutive CaMV 35S Promoter

**DOI:** 10.1371/journal.pone.0167927

**Published:** 2016-12-08

**Authors:** Jitka Viktorova, Zuzana Jandova, Michaela Madlenakova, Petra Prouzova, Vilem Bartunek, Blanka Vrchotova, Petra Lovecka, Lucie Musilova, Tomas Macek

**Affiliations:** 1 UCT Prague, Faculty of Food and Biochemical Technology, Department of Biochemistry and Microbiology, Technicka 3, Prague, Czech Republic; 2 UCT Prague, Faculty of Chemical Technology, Department of Inorganic Chemistry, Technicka 3, Prague, Czech Republic; MJP Rohilkhand University, INDIA

## Abstract

Although stinging nettle (*Urtica dioica*) has been shown to reduce HM (heavy metal) content in soil, its wider phytoremediation potential has been neglected. *Urtica dioica* was cultivated in soils contaminated with HMs or polychlorinated biphenyls (PCBs). After four months, up to 33% of the less chlorinated biphenyls and 8% of HMs (Zn, Pb, Cd) had been removed. Bacteria were isolated from the plant tissue, with the endophytic bacteria *Bacillus shackletonii* and *Streptomyces badius* shown to have the most significant effect. These bacteria demonstrated not only benefits for plant growth, but also extreme tolerance to As, Zn and Pb. Despite these results, the native phytoremediation potential of nettles could be improved by biotechnologies. Transient expression was used to investigate the functionality of the most common constitutive promoter, CaMV 35S in *Urtica dioica*. This showed the expression of the *CUP* and *bphC* transgenes. Collectively, our findings suggest that remediation by stinging nettle could have a much wider range of applications than previously thought.

## Introduction

The long-term contamination of agronomically important soil remains a problem. Some contaminants, especially heavy metals, occur naturally through processes such as the weathering of rocks. However, most are the result of human activities, such as the mining, processing and smelting of ore, and the nuclear and automotive industries. The release of contaminants disrupts the normal biogeochemical balance by their concentration in the environment. Because current remediation technologies, based on physical-chemical processes, have several limitations [1], new methods have to be found. One such remediation technology is phytoremediation.

Phytoremediation, a technology that uses plants for the removal of pollutants from the environment, is an effective, low-cost tool for the degradation of organic compounds or accumulation of heavy metals [2]. Various plant species have mechanisms for the detoxification of xenobiotic compounds, with some being tolerant to high concentrations of toxic compounds and able to hyperaccumulate up to 1% of their weight. Despite its advantages, phytoremediation has numerous drawbacks [3], including low biomass production, short plant roots and difficulties in controlling the growth of hyperaccumulators. These limitations, as well as broad plant substrate specificity, can only be overcome by the use of microorganisms.

The microbial population provides a large reservoir of detoxification genes [4], because microorganisms are evolutionarily adapted to use diverse catabolic pathways to utilize various compounds as energy sources. The transfer of microbial degradation genes to plant species has been shown to be a promising tool. Genetically modified plants bearing microbial genes have been successfully applied in the remediation of soil contaminated by polychlorinated biphenyls [5, 6], explosives [7], pesticides [8] and heavy metals [9–11], amongst other contaminants. Genetic manipulation is not the only way to benefit from the degradation capacity of microorganisms, another way is based on using bacteria with plant growth promoting abilities for the colonization of plant tissues.

Such endophytic bacteria are usually resistant to high concentrations of pollutants and promote plant growth and remediation [12]. Moreover, the cultivation of plants with increased tolerance to pollutants improves the colonization and diversity of surrounding contaminated areas, which are otherwise sparsely populated by both plants and microbes. And finally, higher diversity usually leads to higher remediation.

Phytoremediation has been tested using various plant species and their effect on both inorganic and organic pollutants. To date, around 450 heavy metal hyperaccumulating species belonging to 45 families have been identified [13]. One such reported hyperaccumulating plant is stinging nettle (*Urtica dioica*) [14], which is spread worldwide in mild climate regions, and its growth is associated with human activities. However thus far, no study has investigated the use of HM hyperaccumulator stinging nettle for the phytoremediation of organic compounds. In this study, we cultivate common nettles in real soils that were long-term contaminated with polychlorinated biphenyls (PCBs) and with heavy metals. Both soils originated from dumpsites. Therefore we are focused on determining the natural remediation capabilities of stinging nettle in both soils and the benefits of endophytic bacteria. We report the way how to stimulate native phytoremediation by utilizing the common procedures for the preparation of transgenic plants. Our results show that stinging nettle does not have satisfying phytoremediation abilities, however, they can be improved by agrobacterial infiltration and the expression of genes previously reported to improve phytoremediation.

## Materials and Methods

### Cultivation of nettles in contaminated soils

Plants of *U*. *dioica* were cultivated in pots with two types of contaminated soil. The first soil was collected from the dumpsite of a long-term PCB-contaminated soil in Lhenice, Czechia [15] (49.0°N, 14.2°E). The second soil was obtained from mining ore at Pribram (Czechia) with excessive levels of As, Cd, Pb and Zn (49.7°N, 14.0°E). No specific permissions were required for the access and sampling of the location used, nor did the field study involve endangered or protected species. 20 seeds of *U*. *dioica* were sown into each pot containing approximately 1 l of the genuine contaminated soil. In total six pots were planted for each type of soil, meaning six biological replicas. Nettles were cultivated for four months in a cultivation chamber (Adaptis, Schoeller Instruments) with the following default program: light/dark 8/16 h, 22/20°C and a relative humidity of 30%. Pots were watered three times per week with 40 ml of water. After four months of cultivation, the soils were air-dried at room temperature and passed through a 2-mm plastic sieve.

### Determination of PCB decrease in contaminated soil

The soil from Lhenice was homogenized and 1 g from each replica was extracted into 5 ml of diethyl ether for 6 h while being continuously shaken. The extract was analyzed using a gas chromatograph with a micro-electron capture detector (GC/μECD, Agilent, USA) and evaluated according to Mackova et al. (2009). The residual amount of PCBs in the soil was determined using Agilent ChemStation software (Agilent, USA). The PCB content was determined in three technical replicas. Outlying values were excluded based on the Dixon test (α = 0.05). The results are presented with standard deviations. The data were analyzed using Fisher's Least Significant Difference (LSD) test at 5% probability level.

### Determination of metals decrease in contaminated soil and their amounts in plants

The soil from Pribram was homogenized and 3 g from each replica was shaken overnight in 30 ml of 2M HNO_3_. The extract was then analyzed by flame atomic absorption spectroscopy (Spectr AA880, Varian).

The plant material from each replica was kept separate and split into parts (roots, stems, leaves), frozen with liquid nitrogen, grinded with a pestle and mortar, and lyophilized. Approximately 0.1 g of dried powder was dry ashed over the following temperature gradient for 6 h (160–220–280–350–450–500°C, each temperature was maintained for 1 h). Subsequently, the samples were wet digested by incubating each sample for 1 h at 120°C in 1 ml of concentrated HNO_3_. The digested samples were dry ashed at 500°C for 1 h. The samples were dissolved in 20 ml of 1.5% HNO_3_ for analysis by flame atomic absorption spectroscopy (Spectr AA880, Varian).

### Isolation and characterization of endophytic bacteria

Approximately 0.2 g of plant tissue samples were surface-sterilized for 10 min in 0.1% sodium hypochlorite containing 0.5 ml.l^-1^Tween 20. The sterilized plant parts were washed three times with sterile water. From the last washing, 100 μl of water was transferred onto an agar plate with LB (Luria-Bertani) medium, which served as the control of the sterilization process after 4 weeks of cultivation. The sterile plant parts were macerated in sterile 10 mM MgSO_4_ and crushed with sterile plastic pestles. 100 μl of each sample was inoculated on the agar plate with ½ LB medium as well as their dilutions in the ratio 100× and 10,000×. The plates were cultivated in a 28°C chamber for two weeks. The unique molecular fingerprints of single colonies were measured by Autoflex Speed MALDI-TOF mass spectrometer using direct transfer protocol recommended by manufacturer (Bruker Daltonics). The identification was performed by MALDI Biotyper 3.1 (Bruker Daltonics) equipped with database version 4.0.0.1 containing fingerprints of 5 627 microorganisms. The genus or at least species affiliation was identified. Bacteria non-identified by MS were characterized by sequencing their 16S rRNA using the common primers 8f and 926r, and the method described by [16].

The endophytic bacteria originating from plants cultivated in soil contaminated with PCBs were inoculated into the basal mineral salt solution saturated with biphenyl as the sole source of carbon [16]. The bacteria were cultivated in a 28°C chamber for six weeks. The turbidity, corresponding to bacterial growth, was measured spectrophotometrically each week.

The endophytic bacteria originating from plants cultivated in soil contaminated with metals were inoculated on CAS agar [17] to detect the siderophore production. The plates were cultivated in a 28°C chamber for two weeks, and then the formation of zones was detected. The production of other plant growth promoting factors was tested according to published methodologies: ACC deaminase activity [18], IAA production [19], phosphate solubilization [20] and nitrogenase activity [21].

To determine the 50% inhibitory concentration (IC_50_), the endophytic bacteria was inoculated to the metal-containing medium to a turbidity of 0.4 McFarland units. Culture growth was monitored with the automated growth curve analysis system Bioscreen C (Growth Curves USA) and the data were adapted to the formula T_c_ = T_0_/(1 + exp(*c* − IC_50_)/*b*)), where T_c_ is the culture turbidity at given metal concentration *c*, T_0_ is the turbidity of the culture with no added metal and *b* is the slope of the sigmoidal dose-dependent curve [22].

### Transient expression of genes under the control of CaMV 35S promoter in nettles

The nettle seeds were planted in the soil. When the seedlings were 10 cm high (after 6 weeks), the *Agrobacterium* was infiltrated into the bottom part of their leaves using a plastic syringe. The bacteria inoculum was prepared according to [23]. The preparation of the strains of *Agrobacterium* used for transient expression has been previously published. Briefly, the *Agrobacterium* strain bearing the transgene *bphC* under the control of the constitutive promoter of Cauliflower mosaic virus incorporated into the vector pPCV provides bacteria and plants with ampicillin and hygromycin resistance [24]. The second strain of *Agrobacterium*, with the transgene *CUP* incorporated into the vector pGreen, carried the kanamycin resistance gene in its transferred DNA [25], and provides bacterial cells with both kanamycin and tetracycline resistance. The transgene *bphC* encodes a 2,3-dihydroxybiphenyl 1,2-dioxygenase, which is responsible for the cleavage of an aromatic ring of PCB, and the *CUP* gene encodes metallothionein with a high affinity for heavy metals.

Both transgenes (*bphC* and *CUP*) were under the control of the same typical plant promoter. The concentrations of antibiotics used were 5 mg.l^-1^ for tetracycline, 100 mg.l^-1^ for kanamycin and ampicillin, and finally 15 mg.l^-1^ for hygromycin.

48 hours after agrobacterial infiltration, the leaves were cut and frozen until the RNA was isolated (RNeasy Plant Kit, Qiagen), purified (DNase, NEB; Murine RNase inhibitor, NEB) and finally trancripted to the complementary DNA (ProtoScript AMV First Strand cDNA Synthesis Kit, NEB). The presence of mRNA responding to the transgenes was confirmed by PCR using cDNA as the template and specific primers for the gene *bphC* (F: ATGAGCATCAAGAGCTTGGGTTAT, R: TCACGAATTCCTTCGCACCGACTT) and *CUP* (F: CATCATGGTATGGCTAGCATGACTGG, R: TCATTTCCCAGAGCAGCATGACTTC). KOD Hot Start DNA polymerase (Novagen) was used for the amplification of both transgenes.

## Results and Discussion

To date, nettles have not been randomly used for the remediation of contaminated soils, even if the literature describes their properties to be suitable for these techniques [26]. The main benefit of this plant species is its simplicity in terms of nutrition requirements, moreover, it is a weed species that spreads wildly around roads, canals and human habitations.

The source of the results described below is a cultivation experiment of nettles in two different types of soil contaminated by human activities. The first was polluted with polychlorinated biphenyls, the second with heavy metals. The phytoremediation potential of *Urtica dioica* was determined for both organic and inorganic types of pollutants.

**Fig 1:**
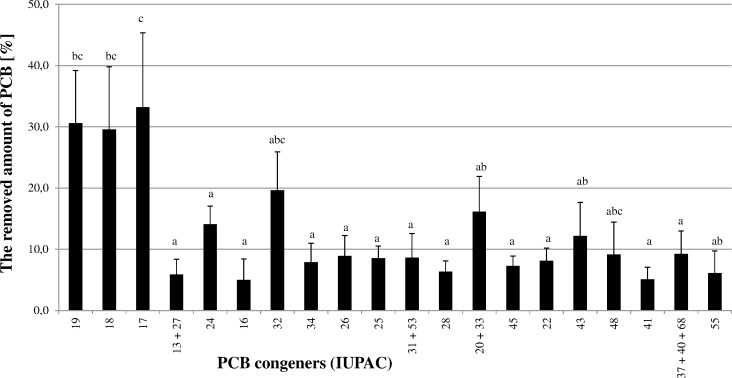
Decrease in PCB congeners caused by planting nettles in the contaminated soil from Lhenice. Standard errors are expressed as a standard deviation depending on the square root of the number of replicas [56]. The decrease in individual congeners was determined as the variance between the congener amount in the planted soil and non-planted soil (expressed in %, relative to 1 g of nettles). The data were analyzed using Fisher's Least Significant Difference (LSD) test at 5% probability level.

The potential of nettle phytoremediation for removing organic compounds has never been tested. This is the first report focused on the remediation of PCB by nettles (Fig 1). The metabolism of different congeners is strongly dependent on plant species, because the molecular configuration plays an important role in PCB metabolism [27]. According to our results, nettles are only able to remediate less chlorinated biphenyls. This is not very surprising, because it was previously shown that the lower chlorination grade is associated with higher metabolism rates [28]. A decrease of up to 33% was determined for trichlorinated biphenyls (congeners 13–39). Up to 12% of tetrachlorinated biphenyls (congeners 40–81) were removed. Other chlorinated biphenyls were hardly removed at all. For pentachlorinated biphenyls, up to 2.4 ± 0.8% (congener 84, 87 and 101) 1.3 ± 0.8% (congener 95, 99 and 110) were removed. Congeners 136, 147 and 134 were the only hexachlorinated biphenyls to be removed (up to 2.7 ± 1.7%). No hepta-, octo or nonachlorinated biphenyls were removed from the planted soil compared to the non-planted soil. Surprisingly, each of the above-mentioned congeners is chlorinated at both the 2 and 2´ positions. The most removed hexachlorinated conger (136) is substituted in each *ortho* position, i.e. positions 2, 2´, 6 and 6´. Similarly, congener 19 (2,2´,6) is one of the best removed congeners, however other congeners substituted in this way (congeners 54, 96 and 104) were not present in the soil. These highly *ortho*-substituted PCB congeners evidently play a role in the toxicities elicited by the PCB mixture by decreasing the dopamine content in the brain [29]. Therefore, even though their overall remediation is not very high, the remediation that they do perform is highly important.

Even if the decrease of PCBs amount in soil is evident, the impact of nettle for removal is still unclear. Usually, the phytoremediation of organic pollutants could be conducted i) in the rhizosphere by soil bacteria whose degradative genes are induced by plant secondary metabolites secreted from plant roots or ii) after the translocation of pollutant into the plant tissue by plant enzymes [30]. To the best of our knowledge, this is to the first report focused on the removal of an organic pollutant in soil by nettle and so far no study focused on rhizodeposition of nettle’s secondary metabolites. Therefore, there is no study available for comparison of stimulation of rhizoremediation by nettle’s released secondary metabolites with many publications focused on the rhizoremediation facilitated by secondary metabolites from numerous plants (e.g. review of [31]). On the other hand, the presence of typical enzymes responsible for detoxification in plant tissue has been several times published in nettles. For example, the transformation of the initial substrates includes enzymatically catalyzed reactions involving enzymes such as P450 monooxygenases, peroxidases, reductases, dehydrogenases and esterases. The presence of peroxidase, polyphenol oxidase and catalase in nettles and their effect on catechol transformation has been previously described [32]. Another research group [33] was focused on glutathione and its related enzymes as a glutathione reductase, glutathione transferase and glutathione peroxidase clarifying the effect of these enzymes on conjugation and detoxification of xenobiotics in nettles. Although the exact mechanism of PCBs detoxification by nettles is still unknown, it is evident, that many general mechanisms for PCBs removal from soil by plants are valid for nettles as well.

The phytoremediation potential of nettles was evaluated according to translocation factor (TF, ratio of heavy metals concentration in shoot and root) [34], biological concentration factor (BCF, ratio of heavy metals concentration in root and soil) [35] and finally, according to biological accumulation factor (BAF, ratio of heavy metals concentration in shoot and soil) [35]. Plant species with a value higher than 1 are regarded as efficient in phytoextraction, phytostabilization and/or phytoaccumution. Based on our results, nettles belong in the group of zinc hyperaccumulating plant species. Zinc is an essential element in many plant proteins and enzymes. Plants have evolved the ability to accumulate and preserve considerable amounts of zinc inside cell vacuoles. Currently, 14 taxa of zinc hyperaccumulating plant species are known, including the best studied *Arabidopsis halleri* [36]. On the other hand, a completely different situation was described for lead, which was mostly uptaken into the root tissues and was hardly translocated to the green plant parts at all. While the TF of zinc was 1.1 ± 0.1, the TF of lead was 0.4 ± 0.1 (Table 1). A similar trend was observed by other research groups [37, 38], who explain it as an antagonistic effect of zinc on the uptake of lead. [38] also reported the same antagonistic effect of zinc on the uptake of cadmium, whose concentration in plants was practically undetectable in our experiment, in contrast to the high concentration of zinc.

**Table 1:** Amount of acid-extractable metals in nettles cultivated for four months in contaminated soil.

	Heavy metals concentration in nettle (mg/kg dw)			Phytoremediation potential		
	Root	Shoot	Leaf	TF	BCF	BAF
**Pb**	108.9 ±6.5	43.3 ± 4.0	5.3 ± 0.6	0.4 ±0.1	0.3 ± 0.0	0.1 ± 0.0
**Zn**	85.1 ± 5.3	95.0 ± 2.6	46.5 ± 0.7	1.1 ± 0.1	0.9 ± 0.1	1.0 ± 0.1

The phytoremediation potential is presented as a translocation factor (TF), biological concentration factor (BCF) and biological accumulation factor (BAF). The results are presented with standard errors.

The decrease in heavy metals in soil was 4.9±0.2, 5.3±0.4 and 19.4±0.8% for lead, cadmium and zinc, respectively (Table 2). To date, phytoremediation by nettles was reported for several metals: As, Cd, Cu, Pb, Zn, Hg, Cu, Cr [26, 39–41] and bioindicating capacity for F, Zn, Fe, Mn [42, 43]. In comparison to our study, Boshoff et al. (2014) found a higher metal content in nettle (Pb = 130 mg.kg^-1^, Zn = 475 mg.kg^-1^). However, the plant accumulation of metals highly depends on the concentration of metals in the soil, and the above-mentioned publication used soil contaminated up to 4 times or 60 times (Pb and Zn, respectively) as much as our soil. Grubor et al. (2008) found a 36% decrease in lead in soil after 3 weeks of nettle cultivation, which is almost incomparable with our 5% decrease after four months. However, their soil was 5 times as contaminated with lead as our soil (Table 2). Arsenic was undetectable in nettles in our study with soil containing almost 10 mg.kg^-1^ of As. Boshoff et al. (2014) found 11 mg.kg^-1^ of As in nettles, however their soil was 14 times more contaminated with As than our soil. Therefore, our lower accumulation corresponds to the soil used, which originated from silver and uranium mining.

**Table 2:** The results of soil analysis in terms of heavy metals before and after four-month nettle cultivation.

(mg/kg dw)	Pb	Cd	Zn	As
**original soil**	354.1 ± 10.6	1.9 ± 0.1	96.7 ± 2.9	9.9 ± 0.3
**vegetated soil**	336.8 ± 0.6	1.8 ± 0.0	77.9 ± 1.0	10.4 ± 0.1

The amount of arsenic was determined to be about 11% higher after the cultivation period. The reason for this arsenic content increase could lie in the fact that only acid-extractable metals are determined. To improve our understanding we have to note that a certain amount of metals is always adsorbed to particulates, and is less likely to enter the solution for analysis. Because the growing plant produces a range of secondary metabolites at its roots, soil properties are usually changed as well as the amount of extractable and bioavailable metals.

**Fig 2:**
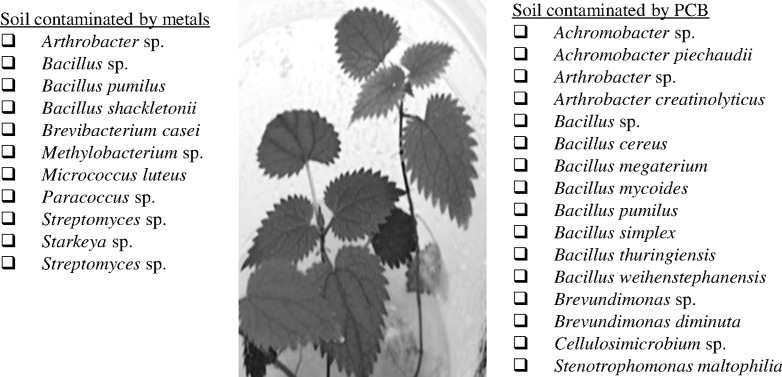
Endophytic bacteria isolated from nettles. Nettles growed in soil contaminated with heavy metals (left) or with polychlorinated biphenyls (right).

The potential of endophytic bacteria to enhance phytoremediation has been previously reported [44]. Therefore, our efforts to support phytoremediation began with studying endophytic bacteria and their contribution to plant health and tolerance to toxic compounds. The same group of bacterial genera was isolated from both types of nettles cultivated in different contaminated soil: *Arthrobacter* sp., *Bacillus* sp. and *B*. *pumilus*. Both bacterial genera were previously identified as endophytes in other plant species, e.g. *Alyssum bertolonii* [45], cotton and corn [46]. Moreover, both types of nettles contained some specific bacteria (Fig 2). Both groups of endophytes were cultivated under selective conditions to find bacteria degrading PCB or producing siderophores for HM accumulation. None of the isolated endophytic bacteria were able to grow in the presence of biphenyl as the sole source of carbon; on the other hand five endophytes were able to create large zones in CAS agar. These endophytes were studied for the production of plant growth promoting (PGP) factors, and *B*. *shackletonii* and *Streptomyces badius* were determined to be the most beneficial for plant growth. *S*. *badius* was found previously in Asteraceae plants [47], however, its PGP have been never tested. The only bacterium to have been previously reported to be an endophyte producing PGP factors is *B*. *pumilus*. Its production of siderophores and inability to dissolve phosphates has been confirmed by several authors [48, 49]. *B*. *shackletonii* was found to be the most resistant bacteria to the presence of heavy metals in the growth medium. This rather novel species was isolated in 2004 from a volcanic soil [50] which is usually rich in heavy metals, like our soil from the mine. Our measured values for IC_50_ (Table 3) correspond to those determined for other HM-resistant bacterial strains [22, 51]. The As-resistance genes were characterized in *Bacillus* species [52] and other mechanisms of resistance were summarized in a comprehensive review [53].

**Table 3:** The plant growth promoting factors (PGP factors) of isolated endophytes producing a high amount of siderophores.

Name	Identification score	PGP factors					IC_50_ (μM)			
		^a^	^b^	^c^	^d^	^e^	As	Cd	Zn	Pb
*Rhizobium mesosinicum*	**0.981**^f^	**Y**	N	**Y**	N	**Y**	628 ±125	**178±7**	**5 463±1 352**	115±7
*Bacillus shackletonii*	**2.189**^g^	**Y**	**Y**	**Y**	**Y**	**Y**	**6 851±367**	**176±150**	**˃6000**	**>200**
*Streptomyces badius*	**1.801**^g^	**Y**	**Y**	**Y**	**Y**	**Y**	1 556±11	89 ±27	**˃6000**	**>200**
*Arthrobacter rusicus*	**0.994**^f^	**Y**	N	**Y**	N	**Y**	3 977±148	35±12	**˃6000**	**>200**
*Bacillus pumilus*	**2.085g**	**Y**	N	**Y**	N	N	**>6000**	13±1	3 946±996	**>200**

The inhibition concentrations of metals, killing 50% of the population, are presented with standard deviation.

^a^ Siderophores production

^b^ IAA production

^c^ ACC-deaminase activity

^d^ Phosphate solubilisation

^e^ Nitrogenase activity

^f^ MS identification score. Bacteria was identified using MALDI-TOF mass spectrometer.

^g^ 16S rRNA similarity. Bacteria was identified using 8F and 1026R primers for sequencing of 16S rRNA.

**Fig 3:**
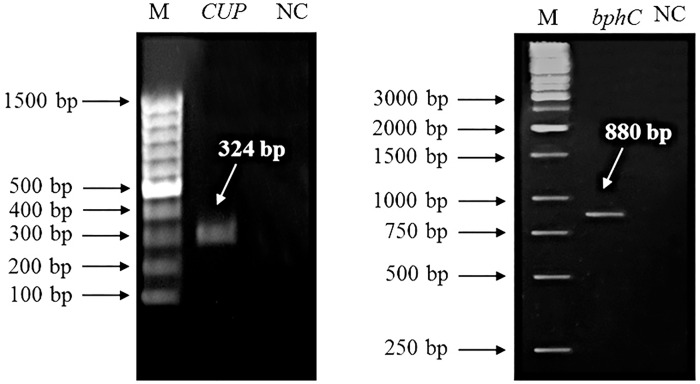
Detection of transgene expression at mRNA level in transiently transformed nettles. The transgenes used, *CUP* (left) and *bphC* (right), originate from microorganisms, where they play an important role in remediation and tolerance to pollutants.

Therefore in summary, endophytic bacteria should improve the resistance of plants to heavy metals by their detoxification mechanisms. Moreover, they play an important role in plant growth promotion, because the endophytic bacteria produce significant small molecules or enzymes, e.g. plant hormones or enzymes providing plant micro- and macronutrients. Therefore, the benefit of PGP by endophytic bacteria should manifest itself in a higher biodiversity of contaminated areas. On the other hand, genetic engineering methods have demonstrated their benefits in phytoremediation [2]. Therefore, this aspect of improving the native phytoremediation potential of nettles could not be omitted. Transgenic nettles have never been prepared before, and a lot of experiments will need to be done before a permanent transgenic nettle can be obtained. To the best of our knowledge, our report is the first to focus on recombinant expression in nettle tissues; therefore we designed our experiment as a transient expression of transgenes in the leaves of this species. The constructs for agrobacterial infiltration to the plant were prepared previously. Both transgenes (*bphC* and *CUP*), playing a significant role in bacterial remediation, were cloned under the control of the strong constitutive promoter of cauliflower mosaic virus (CaMV 35S). Even though the promoter is frequently used for the preparation of transgenic plants, its functionality has to be first verified in each new species, because the CaMV promoter in transgenic DNA differs significantly from the plant´s own promoters and integrated viruses [54]. We performed the transient expression of both remediation genes. The bacterial inoculum was infiltrated into the bottom part of leaf with a syringe, as is commonly done with the plant model species tobacco [55]. The expression of both transgenes was verified at the mRNA level after the isolation and purification of RNA and reverse transcription to DNA by PCR using non-infiltrated nettle plants as a negative control (Fig 3). As was shown, *Agrobacterium* is able to attack the plant cells of nettles and incorporate part of its DNA to the plant genome, and plant polymerases are able to recognize CaMV 35S promoter and transcribe the transgene into the mRNA. Therefore, a lot of previously published vectors could be universally used for the agrobacterial transformation of nettles, as well as our constructs bearing the *bphC* or *CUP* gene, to improve their phytoremediation.

## Conclusions

This paper is the first to take an interest in using nettles for the phytoremediation of not only heavy metals, but also organic compounds. The potential of nettles to remove heavy metals from soil was previously published and confirmed by our research. However, it was demonstrated here that nettles are able to remove up to more than 30 percent of mono- di- and tri-chlorinated biphenyls. Moreover, their endophytic bacteria and their benefits to plant growth and resistance are presented here, as well as the potential of genetic engineering for improving the native phytoremediation of nettles.
