# Correction: Native Phytoremediation Potential of *Urtica dioica* for Removal of PCBs and Heavy Metals Can Be Improved by Genetic Manipulations Using Constitutive CaMV 35S Promoter

**DOI:** 10.1371/journal.pone.0187053

**Published:** 2017-10-19

**Authors:** Jitka Viktorova, Zuzana Jandova, Michaela Madlenakova, Petra Prouzova, Vilem Bartunek, Blanka Vrchotova, Petra Lovecka, Lucie Musilova, Tomas Macek

The following information is missing from the Funding section: This study was supported by the European Union Horizon 2020 Research and Innovation Programme (Grant No. 692195).
